# A status report on RNAi therapeutics

**DOI:** 10.1186/1758-907X-1-14

**Published:** 2010-07-08

**Authors:** Akshay K Vaishnaw, Jared Gollob, Christina Gamba-Vitalo, Renta Hutabarat, Dinah Sah, Rachel Meyers, Tony de Fougerolles, John Maraganore

**Affiliations:** 1 Alnylam Pharmaceuticals Inc., 300 Third Street, Cambridge, MA 02142, USA

## Abstract

Fire and Mello initiated the current explosion of interest in RNA interference (RNAi) biology with their seminal work in *Caenorhabditis elegans*. These observations were closely followed by the demonstration of RNAi in *Drosophila melanogaster*. However, the full potential of these new discoveries only became clear when Tuschl and colleagues showed that 21-22 bp RNA duplexes with 3" overhangs, termed small interfering (si)RNAs, could reliably execute RNAi in a range of mammalian cells. Soon afterwards, it became clear that many different human cell types had endogenous machinery, the RNA-induced silencing complex (RISC), which could be harnessed to silence any gene in the genome. Beyond the availability of a novel way to dissect biology, an important target validation tool was now available. More importantly, two key properties of the RNAi pathway - sequence-mediated specificity and potency - suggested that RNAi might be the most important pharmacological advance since the advent of protein therapeutics. The implications were profound. One could now envisage selecting disease-associated targets at will and expect to suppress proteins that had remained intractable to inhibition by conventional methods, such as small molecules. This review attempts to summarize the current understanding on siRNA lead discovery, the delivery of RNAi therapeutics, typical *in vivo *pharmacological profiles, preclinical safety evaluation and an overview of the 14 programs that have already entered clinical practice.

## Introduction

Since the original reports of RNA interference (RNAi) in cells from a range of species [1-3], there has been increasing interest in harnessing this endogenous mechanism, which enables degradation of a specific mRNA, as a novel pharmacological approach to human disease. Indeed, from a drug discovery perspective, small interfering (si)RNAs have some distinct advantages over conventional drug therapies such as small molecules or antibodies (Table 1). However several major obstacles have had to be overcome before the entry of RNAi therapeutics to clinical trials. These include steps required for lead selection, the use of chemical modifications to confer appropriate biopharmaceutic properties, the design of formulations that enable delivery to a target tissue, and screening of these products for safety, including assessments for potential off-target effects. These aspects are addressed below and followed by a critical analysis of the 14 programs that have entered clinical development in the past decade. This review does not cover the related and rapidly expanding field of RNA therapeutics, which addresses microRNAs (miRNAs) rather than messenger mRNAs, as targets.

**Table 1:** A comparison of various drug discovery attributes of siRNAs and small molecules

	siRNA	Small molecule
Specificity	High, sequence driven	Low-medium, conformation driven

Potency	Typically pM	Variable

Number of accessible targets	>>1000	500 to 1,000

Number of potential leads and backups	>>10 to 100, depending on length of target	<2 to 3

Speed to lead molecule	4 to 8 weeks	2 to 4 years

Species crossreactivity	High	Low

Manufacturing	Common, rapid, scalable methods	Variable, can be complex

### Lead discovery algorithms

**Figure 1:**
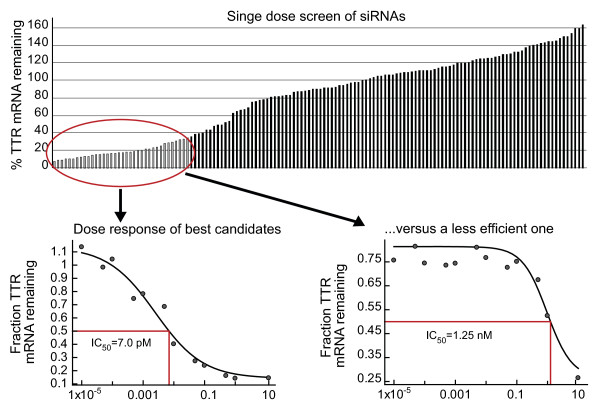
**Small interfering (si)RNA Lead selection**. A large panel of siRNAs identified by a bioinformatic screen were synthesized and tested *in vitro *for activity against the transthyretin transcript as measured by quantitative PCR. The upper part of the panel shows the entire panel tested in parallel at a given nanomolar siRNA concentration, and data are shown in rank order of potency, with each vertical line representing an individual siRNA. The bottom panel illustrates *in vitro *dose response curves for a potent versus a less potent molecule.

We and others have developed high-throughput algorithms to support screening and selection of a lead siRNA. De Fougerolles *et al*. [4] reviewed the various steps involved, which include a bioinformatic screen to identify duplexes 19-23 bp in length with minimal off-target complementarity, small-scale synthesis of a panel of siRNAs, *in vitro *assays for potency and nonspecific cytotoxicity, and assessment of *in vivo *pharmacology. Embedded in this primary screen are subscreens for stability in a biological matrix (for example, serum, cerebrospinal or bronchoalveolar lavage fluid) relevant to the target tissue, and additional screens for specificity (addressed below). The ubiquitous nature of RNases requires that in most cases, a lead siRNA needs to be stabilized. Increased stability can be achieved by chemically modifying the primary sequence. To date, the modifications used have been phosphodiester to phosphorothioate modifications on the backbone and 2"-*O*-methyl or other 2" substitutions on the ribose moieties [5]. This strategy has been influenced by the antisense oligonucleotide (ASO) field, in which both modifications have been used extensively and which provides an accompanying body of safety data in preclinical species and in humans. Many additional modifications have been explored both for siRNAs and ASOs [5], including locked nucleic acids, in which the 4" carbon on the sugars is tethered to the 2" substituent, although fewer molecules of this type have to date reached clinical trials. For any given sequence, the number and position of the chemical modifications necessary is variable and requires an empiric approach. Given that chemical modifications, especially to the antisense or 'guide' strand of the duplex, can influence potency, each round of modifications also requires a secondary screen to determine maintenance of activity. However, at the end of this type of screening process, lead siRNAs with IC50 values in the low single-digit picomolar to femtomolar range can often be identified (Figure 1). The combination of attractive mechanism of action, efficiency of siRNA lead discovery and relative ease of siRNA manufacture explain why both biotechnology and pharmaceutical companies have shown great enthusiasm for RNAi therapeutics [6].

### Delivering RNAi therapeutics

The ~14 kDa mass and polyanionic charge of a typical 21 bp siRNA duplex ensure that achieving good tissue bioavailability is often a greater challenge than lead selection. Despite this, both local and systemic delivery to various tissue and cellular compartments have been demonstrated preclinically [4,7,8]. For local delivery, administration of unmodified siRNA in simple formulations such as saline has resulted in target mRNA knockdown in a wide variety of tissues, including the respiratory and urogenital epithelia, central nervous system and the eye. Most of the local delivery studies have been in mice, and involve substantial doses, presumably associated with high local concentrations that drive cellular uptake. Although the exact mechanism of uptake in most of these cases remains unknown, some notable effects have been observed, including decreasing respiratory syncitial virus (RSV) or parainfluenza virus (PIV) infection in respiratory epithelium [9,10], herpes simplex virus-2 infection in the vaginal epithelium [11], 2',3'-cyclic nucleotide 3'-phosphodiesterase expression in oligodendrocytes [12], huntingtin expression in neurons [13], and vascular endothelial growth factor (VEGF)-A [14] and VEGF receptor (VEGFR)I [15] expression in ocular tissues.

Systemic delivery of RNAi therapeutics offers both the greatest opportunities and challenges. An unmodified saline-formulated siRNA injected intravenously is subject to simultaneous RNase-mediated degradation and rapid renal excretion. Hence, any attempts at systemic delivery must involve mechanisms for increasing the circulation half-life (t_1/2_) of the siRNA, its distribution to an appropriate tissue compartment and then its uptake, followed by intracytoplasmic release and activity. In this regard, the greatest success to date has been achieved with respect to hepatic delivery, with three distinct approaches, each involving a conjugated or formulated siRNA, warranting review.

**Figure 2:**
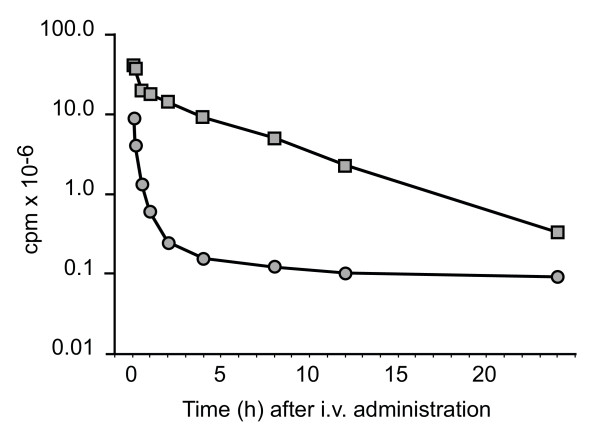
***In vivo *pharmacokinetic profile of a cholesterol-conjugated (squares) and unconjugated (circles) radio-labeled small interfering (si)RNA against ApoB in the mouse **[16]. The half-life and clearance were calculated to be 95 minutes and 0.5 mL/minute and 6 minutes and 17.6 mL/minute, for the conjugated and unconjugated molecules, respectively.

Soutschek *et al*. [16] were the first to report a delivery strategy with significant translational potential. In a mouse system, they demonstrated that cholesterol conjugation to the sense or 'passenger' strand of an ApoB-specific siRNA administered intravenously resulted in a significant reduction in clearance and an associated 16-fold increase in t_1/2_, relative to the unconjugated control (Figure 2). Subsequent work elucidated that the change in pharmacokinetic (PK) characteristics was secondary to loading of the conjugated siRNA into circulating lipoprotein particles via the appended cholesterol moiety [17]. This also facilitated receptor-mediated uptake of the siRNA into hepatocytes, and resulted in ~60% knockdown of ApoB mRNA in the mouse liver [16]. Notably, intravenous administration of cholesterol-conjugated siRNAs targeting ApoB also resulted in efficient knockdown of ApoB mRNA in the jejunum. More recently, conjugation-based approaches with other ligands have been used, such as prostate-specific membrane antigen aptamer-siRNA conjugates to deliver drug to tumor cells *in vivo *[18].

Two groups have reported liposomal nanoparticle (LNP)-mediated delivery of siRNA to the liver, demonstrating knockdown in the mouse [19] and the non-human primate [20]. In both studies, the LNP formulation, also known as a 'stable nucleic acid lipid particle' (SNALP), was composed of several non-covalently associated components (an ionizable lipid, polyethylene glycol (PEG)-lipid for prolonging t_1/2_, cholesterol, and a neutral lipid) which self-assembled and encapsulated the siRNA. Morrissey *et al*. [19] were able to show that relative to unencapsulated siRNA, LNPs that encapsulated a variety of anti-hepatitis B virus (HBV) siRNAs showed reduced plasma clearance and efficient hepatic uptake with dose-dependent, potent (>1 log_10_) and durable knockdown of circulating HBV DNA levels in a mouse model of infection. Zimmermann *et al*. [20] also demonstrated hepatic uptake of LNPs with specific knockdown of ApoB in the non-human primate. The pharmacodynamic (PD) profile in this study was notable not only for the extent of hepatic ApoB mRNA knockdown (>80%), but also for its translation to systemic lowering of low-density lipoprotein cholesterol (LDLc) (by 82%) which persisted for several weeks after a single dose. The latter observation was particularly interesting because the drug was undetectable in the liver of animals beyond 48 hrs, suggesting an apparent PK-PD hysteresis. Recent work with more sensitive quantitative PCR assays has clarified that in fact the drug is present (and presumably stabilized in RISC) at all time points when PD is observed after LNP-mediated siRNA delivery [21] (Alnylam Pharmaceuticals Inc., unpublished observations, Renta Hutabarat). The mechanism of ionizable LNP-mediated delivery has been dissected, and *in vivo *it involves the opsonization of the LNP by ApoE in the circulation, LDL receptor-mediated uptake of the opsonized particles by hepatocytes, and then endosomal release of siRNA into the cytoplasm (Alnylam Pharmaceuticals Inc.) [22].

The third systemic delivery approach of note involves multicomponent complexes. Rozema *et al*. [23] studied particles composed of siRNA conjugated with a dynamic polyconjugate and complexed with PEG to extend t_1/2_, plus a liver targeting ligand, *N*-acetyl galactosamine (NAG). This strategy was successfully used in rodents and non-human primates to show potent and durable hepatic ApoB knockdown with associated reduction in circulating LDLc. Mechanistically, the role of NAG was crucial, as its replacement with mannose abrogated uptake by hepatocytes and instead directed delivery to other hepatic cellular compartments such as Kupffer cells. Bartlett *et al*. [24] used a similar multicomponent concept but complexed a ribonucleotide reductase (RRM2) siRNA to cationic cyclodextrin, along with PEG and a targeting ligand, transferrin. In this system, the transferrin allowed delivery to extrahepatic sites, and target mRNA knockdown was achieved in a subcutaneous tumor xenograft.

Of the three hepatic delivery concepts above, the LNP-mediated approach has received the most attention. Recently, optimization of LNP structure and function has resulted in potent *in vivo *knockdown at doses as low as 0.01 mg/kg [25] (whereas in local delivery (see above) doses are typically >1 mg/kg). LNP-mediated delivery of RNAi therapeutics has now been applied to several different target mRNAs other than ApoB and has been described in five species including the mouse, rat, hamster, guinea pig and non-human primate [20,25-28] (Alnylam Pharmaceuticals Inc., unpublished observations). In terms of pharmacology, the LNP experiments have fully validated the drug-like behavior of siRNAs. For all the LNP studies cited, there is a consistency of observations with respect to PD onset, which generally occurs within 24 hrs with peak effects at 48-72 hrs; duration, which lasts several weeks depending on the potency (and perhaps stability) of the siRNA concerned; strict dose dependency; and return of PD to baseline. Akinc *et al*. [27] also elegantly showed reproducibility of effects with multiple treatment cycles by giving a LNP-formulated Factor VII siRNA once monthly. Many studies have also confirmed the RNAi mechanism of action *in vivo *by demonstrating (via 5" rapid amplification of cDNA ends assay) the anticipated cleavage site in the target mRNA and the lack of effect by identical LNP-mediated delivery of an irrelevant siRNA [20,26-28]. Overall, the potency, predictable and reproducible pharmacology across different targets and species (as anticipated for an endogenous mechanism), the selectivity and the clear mechanism of action suggest a robust translational potential for LNP-based hepatic delivery of RNAi therapeutics.

To fully exploit the therapeutic potential of RNAi, systemic delivery beyond the liver will need to be accomplished, and indeed some creative approaches have been reported. With optimization to further increase the circulation t_1/2 _relative to LNPs delivering to the liver, LNP-mediated extrahepatic delivery to subcutaneous tumors in mice has been achieved [29]. Antibodies facilitating siRNA delivery offer several advantages, including an intrinsic targeting mechanism and good PK properties. Antibody-protamine fusion proteins have been shown to complex with siRNAs and reported to deliver specifically to subcutaneous tissue [30] and to lung [31] tumors in mice. By bringing together the advantages of LNPs and antibodies, Peer *et al*. [32] devised sophisticated neutral LNPs with a covalently attached antibody against β7 integrin and carrying an anticyclin D1 siRNA complexed with protamine. Using this approach, they were able to show specific knockdown of cyclin D1 in gut mononuclear leucocytes with translation to therapeutic effects in a mouse model of colitis. Oral delivery of RNAi therapeutics would offer the greatest convenience if translatable clinically. Recently, glucan-encapsulated siRNA particles were reported to be efficient oral delivery vehicles to target gut macrophages [33]. Although the extrahepatic delivery concepts above demonstrate progress, further work is required to reproduce the observations and demonstrate a clear RNAi-dependent mechanism of action, much as described for extrahepatic delivery to subcutaneous tumors [29].

### Safety screening: in vitro

**Figure 3:**
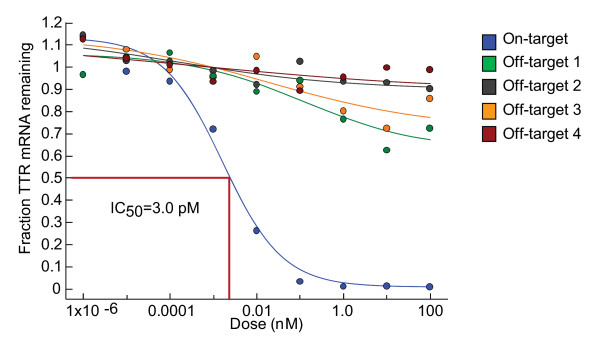
**Comparison of on- and off-target effects**. A putative lead molecule was tested *in vitro *to evaluate potency against the intended target, transthyretin and four sequence-related off targets defined by the bioinformatic screen. The percentage reduction in transythyretin levels was measured by quantitative PCR.

Several *in vitro *studies over the past decade have suggested that RNAi therapeutics have the potential for off-target effects [34]. These are potentially of three types: sequence-dependent/RISC-mediated [35-37], sequence-independent/RISC-mediated [38,39] and sequence-independent/innate immune-mediated [40-42]. The first, produced by binding of the sense or antisense strand to bystander mRNA(s), might lead to an RNAi-mediated off-target effect, or if hybridization occurs in the 3' untranslated region (UTR) then potentially to miRNA-like translational suppression. Bioinformatics, of course, plays a crucial role in minimizing these potential liabilities by deprioritizing sequences that have significant off-target complementarity. Rarely, sequences of interest that have high off-target potential cannot be avoided. In those instances the relative on-target versus off-target half-maximal inhibitory concentrations (IC50s) should be determined *in vitro *(much as for small molecules). We have found that this approach generally reveals that, despite high degrees of off-target complementarity, *in vitro *potency is clearly distinguishable with IC50s for on- and off-target effects separated by several log orders (Figure 3).

Notwithstanding the utility of a robust bioinformatic screening algorithm, multiple studies have detected widespread transcript dysregulation [34]. Most of the studies have relied on *in vitro *siRNA transfection followed by transcriptome readouts. These types of datasets require cautious interpretation for several reasons, including the lack of clear cause and effect relationships (that is, how much of the transcriptional dysregulation is secondary to on-target versus off-target knockdown), the relatively modest degree of transcript dysregulation described (typically an increase or decrease of ≤ two-fold) and the lack of protein or functional readout. When protein correlates have been examined, the siRNA-associated off-target effects have resulted in either very subtle effects or in a lack of quantitative relationship between change in bystander mRNA and protein expression [43]. Finally, studies examining off-target effects have used molecules with suboptimal potency and specificity requiring suprapharmacologic doses to achieve target knockdown. For example, Bilanges and Stokoe [44] observed significant off-target effects with ASO- and siRNA-based approaches against a specific target, phosphoinositide-dependent kinase (PDK)1; however, they used high, potentially cytotoxic concentrations (>300 nM, in contrast to the pharmacologic (picomole to femtomole) levels discussed above (Figure 1).

From a drug development viewpoint, it is widely acknowledged that many molecules, including ASOs [45] and small molecules, from acetaminophen [46] to kinase inhibitors [47], all show diverse effects on transcriptional profiling analyses. Nevertheless, preclinical transcriptional profiling has not been routinely used in lead selection, as these effects have not been shown to have any *in vivo *consequence in terms of safety or efficacy.

**Figure 4:**
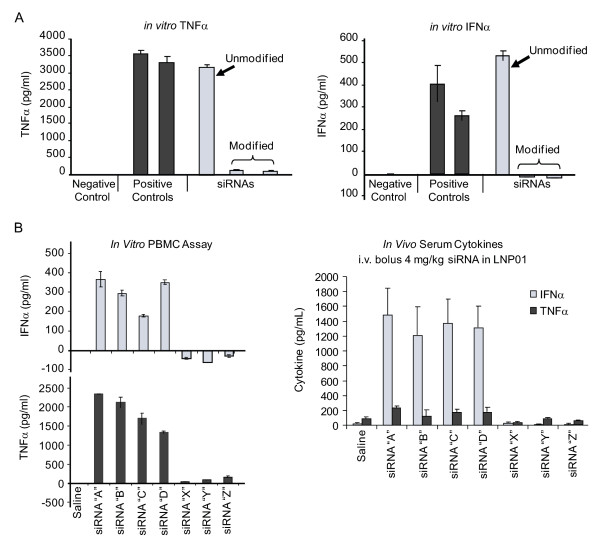
**Profiling immunostimulatory small interfering (si)RNAs**. **(a) **A panel of siRNAs including negative and positive controls were evaluated in an *in vitro *human peripheral blood mononuclear cell (PBMC) assay as described previously [10] with supernatants examined for tumor necrosis factor (TNF) (left panel) and interferon-α (right panel) levels. The right-hand side of each panel has a chemically unmodified siRNA, which is compared with the same sequence after incorporation of a combination of phosphorothioate and 2'-*O*-methyl chemical modifications. **(b) **The left-hand panels show a series of immunostimulatory (A-D) and non- immunostimulatory (X-Z) siRNAs evaluated in an *in vitro *PBMC assay. The right-hand panel shows plasma cytokine profiles in mice injected intravenously with the same siRNAs formulated in LNP01 [26]. Common immunostimulatory siRNAs are identified by the *in vitro *and *in vivo *assays.

Sequence-independent off-target effects refer to either saturation of the endogenous RISC machinery [38,39] or to the immunostimulatory potential of siRNAs [48]. Single-stranded and double-stranded RNAs, particularly chemically unmodified sequences, can stimulate the innate immune system via Toll-like receptor (TLR)-3 [14], TLR-7/8 [40-42] and non-TLR pathways, such as retinoic acid inducible gene (RIG)-I [49] or PKR [50]. Cytokine induction can contribute to target suppression via an RNAi-independent mechanism [14,51]. Eliminating this proinflammatory liability is therefore crucial from both a safety and efficacy perspective. Tractable *in vitro *[10,40] and *in vivo *[41,52] preclinical assays exist, which can assess the proinflammatory potential of an siRNA. Similar approaches were validated when proinflammatory DNA oligonucleotides acting as TLR-9 agonists were studied preclinically and then in clinical practice [53,54]. From a practical viewpoint, because there is no *a priori *knowledge as to which pathway might be engaged for any candidate siRNA, a wide range of inflammatory markers should be evaluated during lead selection, including type I and II interferons, and cytokines and chemokines induced by TLR-3, TLR-7 and TLR-8 agonists. The use of appropriate control siRNAs is crucial in immunostimulatory screening assays, and a widely used, chemically modified green fluorescent protein (GFP) sequence has served as an important negative control for many groups [51]. In certain cases, a given sequence with proinflammatory potential might be very desirable for potency or other considerations. Fortunately, both increased stability and reduced pro-inflammatory liabilities can be achieved simultaneously by chemical modifications (Figure 4). Again, the process of modification is empiric and requires subsequent confirmation that potency has been maintained. It is to be hoped that greater knowledge of TLR recognition will enhance bioinformatic exclusion of proinflammatory sequences and guide chemical modification strategies to specific motifs [40,41,55], increasing overall screening efficiency and throughput.

### Safety screening: in vivo

The entry of an RNAi therapeutic program into clinical practice requires completion of good laboratory practice (GLP)-compliant preclinical toxicological studies. Studies in the rat and dog are used preclinically for the vast majority of drugs destined for clinical practice. By contrast, ASO programs set a precedent by using mouse and non-human primate [56]. It is our understanding that all RNAi therapeutics currently in clinical trials used a rodent and non-human primate as preclinical toxicological species. The reasons underlying this choice are the same for ASO and RNAi programs, and include the availability of bioinformatic databases to enable design of a single crossreactive siRNA for use in rodent, non-human primates and humans; the ability to leverage prior animal pharmacology work with the relevant siRNA; the availability of tools and assays such as immunological readouts and knockout mice; and finally the hope that the non-human primate represents the closest physiology to that of humans.

Some principles regarding the design of preclinical toxicology studies for RNAi programs are now discussed. In both test species, low, medium and high exposures should be achieved using the low dose at or near the anticipated pharmacological dose and the highest dose to represent a significant excess, allowing definition of a maximum tolerated dose. Dosing schedule and timing of terminal sacrifice should take into account the duration of pharmacologic effect, given that single intravenous doses can exert target mRNA knockdown for several weeks, as discussed above. If the candidate siRNA is not fully complementary to the target mRNA in both preclinical species, then a sequence should be selected that is crossreactive to at least one preclinical species, allowing study of on-target toxicity secondary to target mRNA knockdown. For systemic delivery programs, adequate controls should be considered including, where appropriate, comparison of formulated candidate siRNA, formulated irrelevant siRNA and naked (unformulated) siRNA, in order to dissect the relative contributions of siRNA and formulation to the toxicological profile. Beyond the safety endpoints that would be expected in a GLP toxicological program, additional readouts of interest include cytokines and chemokines, specifically those relating to immunostimulation-related effects via TLR activation (see above). Currently, it is not known which preclinical species is most predictive of immunostimulation-mediated effects but a combination of *in vitro *screening along with *in vivo *readouts in two species before clinical trials are held is an appropriately conservative approach. Finally, suitable high-sensitivity bioanalytical assays need to be incorporated to track the siRNA [57], formulation components (if they contribute to the overall profile) and associated metabolites.

Details of GLP toxicology programs are rarely published, and few data are in the public domain. However, no evidence to suggest toxicities similar to those seen with first-generation ASO [56] has emerged. Accordingly, there are no reports of siRNA-related complement activation, coagulation defects, immunostimulation and lymphoid hyperplasia or renal tubular changes. We and others have studied unformulated siRNAs at doses >100 mg/kg in the rodent and non-human primate and found them to be well tolerated, although this is in all likelihood secondary to the rapid renal elimination of naked (unformulated) siRNAs. For systemic delivery formulations such as LNPs and multicomponent complexes with approximate diameters of 50-110-15 nm, respectively, particle size might be an important determinant of biodistribution and therefore target organs for toxicity. For these types of nanoparticles, vascular egress would be expected predominantly in tissues with fenestrated (~100 nm apertures) microvasculature, such as liver, spleen and bone marrow. Consistent with this and the substantial blood flow through the liver, hepatic transaminase elevations were reported in association with LNPs [20] and multicomponent complexes [24]. Abrams *et al*. [21] studied LNP201, a cationic LNP encapsulating an anti-SSB siRNA. They demonstrated that LNP201 localized predominantly to liver and spleen, and with the use of appropriate controls found that the formulation (and not the siRNA) had proinflammatory properties and at high doses was associated with hepatotoxicity. The latter could be abrogated by corticosteroid pretreatment. Thus, the liver might come to be recognized as an important target organ of toxicity as we learn more about systemic delivery formulations.

### Clinical RNAi pipeline

A total of 14 RNAi therapeutic programs have entered clinical practice in the past decade (Table 2). Currently few publications detailing these clinical experiences have emerged, thus much of the information that follows has had to be gleaned from corporate press releases or the US National Institutes of Health hosted database of ongoing and completed clinical trials http://www.clinicaltrials.gov. Of the 14 programs, seven involve local/topical delivery to the eye (four), respiratory tract (two) and skin (one). The remaining seven are systemic programs targeting liver (two), hepatic and extrahepatic cancer (three), leukocytes (one) and kidney (one). All 14 programs are being developed for indications with a high degree of unmet medical need. A review of previous oligonucleotide programs reveals that the ASO field was hampered until recently by a lack of clear proof of concept in humans, largely because of two factors: poor target validation and lack of clear PD readouts in early clinical development [58]. There is significant variability in the degree of prior target validation across the global RNAi pipeline. Six of the 14 programs (ALN-RSV, ALN-VSP, ALN-TTR, TD101, bevasiranib, Bcr-abl) have clear target validation, and the other eight programs address targets of significant interest (for example, p53 for kidney injury or RTP801 for age-related macular degeneration (AMD)), but lack robust prior validation. Equally, only a few programs (ALN-TTR, ApoB-SNALP) offer the possibility of early PD demonstration of target knockdown, whereas the rest will require further development into phase II before adequate proof of concept is achieved. ALN-RSV has achieved initial proof of concept, and is discussed separately below.

**Table 2:** The global RNA interference pipeline*

Sponsor	Program (clinical stage)	Status	Target	Indication	Number enrolled
Alnylam/Cubist/Kyowa Kirin	ALN-RSV (phase IIb)	Ongoing	RSV nucleocapsid	Adult RSV infection	354

Pfizer/Quark	PF-04523655 (phase II)	Ongoing	RTP801	(1) AMD, diabetic macular edema	244†

Quark	QPI 1002 (phase II)	Ongoing	p53	(1) Acute kidney injury, delayed graft function	56†

Zabecor	Excellair (phase II)	Ongoing	Syk kinase	Asthma	?

Alnylam	ALN-VSP (phase I)	Ongoing	VEGF, KSP	Primary and secondary liver cancer	55†

Calando	CALAA-01 (phase I)	Ongoing	RRM2	Cancer	36†

Silence	Atu-027 (phase I)	Ongoing	PKN3	Cancer (GI, lung other)	33†

Sylentis	SYL040012 (phase I)	Ongoing	β2 adrenergic receptor	Glaucoma	?

Alnylam	ALN-TTR (phase I)	Ongoing	TTR	TTR amyloidosis	Enrollment begins H1, 2010

Opko	Bevasiranib (phase III)	Terminated	VEGF-A	AMD	522

Allergan/SIRNA	AGN211745 (phase II)	Terminated	VEGFRI	AMD	164

Tekmira	ApoB SNALP (phase I)	Completed	ApoB	Hypercholesterolemia	23

Transderm	TD101 (phase I completed)	Completed	Mutant K6a	Pachyonychia congenita	1

Univ. Duisberg-Essen‡	Bcr-abl (phase I completed)	Unknown	Bcr-abl oncogene	CML	1

AMD = age-related macular degeneration; CML = chronic myeloid leukemia; GI = gastrointestinal; KSP = kinesin spindle protein; PKN = protein kinase N3; RRM2 = ribonucleotide reductase M2; RSV = respiratory syncytial virus; RTP = ; TTR = transthyretin; VEGF = vascular endothelial growth factor; VEGFRI = vascular endothelial growth factor receptor I.

*All data from corporate websites, press releases and http://www.clinicaltrials.gov

†Enrollment ongoing

‡From Koldehoff et al., 2007

Table 2 shows how RNAi therapeutic programs demonstrate some of the unique advantages of the platform. Several have targets (RSV (N) nucleocapsid protein, p53, TTR, ApoB, K6a) that would be 'undruggable' by small molecules or protein moieties. The attempt to target Bcr-abl and mutant K6a shows the potential for allele-specific knockdown. Finally, ALN-VSP builds on data from animal models in which five transcripts were suppressed in parallel [28] by simultaneously targeting two transcripts, VEGF and kinesin spindle protein (KSP), a clear advantage in complex indications such as cancer.

From an overall safety perspective, almost 1500 patients and healthy volunteers will soon have been enrolled onto RNAi clinical programs. In total, 1065 have already been studied across all phases of clinical development, including 522 in the bevasiranib phase III studies. These numbers are important because a unique siRNA-related adverse event occurring at a high incidence (for example >10%), would probably already have been identified. Equally, no data have emerged to suggest serious adverse events linked to siRNA exposure, and none of the programs have been placed on 'clinical hold' by regulatory agencies, a probable outcome if untoward safety events were being uncovered. The largest fraction of the safety experience (1284 subjects) relates to the three ocular programs (bevasiranib, PF-04523655 and AGN211745) and to the respiratory program (ALN-RSV), which suggests good local tolerability to RNAi therapeutics.

ALN-RSV (Alnylam Pharmaceuticals Inc., Cambridge, MA, USA) in phase 2b is currently the most advanced program in the global RNAi pipeline. It uses an unmodified siRNA formulated in saline targeting the RSV N gene transcript. Three phase I studies (two intranasal [59] and one inhalational [60]) showed safety and tolerability at doses up to 3 mg/kg. In 2008, this program reported the first initial human proof of concept for an RNAi program. In a double-blind, randomized, placebo-controlled study (*n *= 88) of safety and efficacy, prophylactic intranasal treatment with ALN-RSV was shown to decrease the incidence of experimental upper respiratory tract infection with RSV [60]. Since then, another double-blind, randomized, placebo-controlled phase II study (*n *= 24) has been completed with inhaled ALN-RSV in lung transplant recipients naturally infected with RSV. The findings demonstrated improved symptom scores and lung function in favor of ALN-RSV, and the program will shortly start a phase IIb study in the same indication [60]. The ALN-RSV program also illustrates the stability and compatibility of RNAi therapeutics with devices, because the inhalational delivery requires a nebulizer, and extensive evaluation has demonstrated structural and functional integrity of the drug before and after nebulization.

The drug PF-04523655 (Pfizer, NYC, NY, USA)is administered via the intra-ocular route and targets RTP801, a pro-angiogenic factor. It is currently in phase II development for wet AMD [61] and diabetic macular edema [62]; no further details are yet available. QPI-1002 (Quark Pharmaceuticals Inc., Fremont, CA, USA) is a systemically administered siRNA targeting p53, which is also in phase II development [63,64]. Two studies evaluating the safety of QPI-1002 in different patient populations have been completed [65]. These include a phase I study for the prevention of acute kidney injury in patients undergoing major cardiovascular surgery, and the first part of a phase I/II study in renal transplant recipients for the prevention of delayed graft function. Both PF-04523655 and QPI-1002 are thought to be in simple formulations, but the exact details are not known. The three cancer programs, ALN-VSP (Alnylam Pharmaceuticals Inc.) [66], CALAA-01 (Calando Pharmaceuticals, Pasadena, CA, USA) [67] and Atu-027 (Silence Therapeutics, London, UK) [68] are all systemic delivery approaches that are in phase I studies. ALN-VSP and Atu-027 rely on LNP-mediated delivery [60,69] whereas CALAA-01 deploys a multicomponent complex delivery system [25]. All three programs are at early stages and no data have yet been released, although preliminary ALN-VSP data are expected by mid 2010. No data are available for the asthma program, Excellair (targeting syk kinase), other than that in phase I, a total of 21 daily inhalational doses were well tolerated [70].

Most recently, Tekmira Pharmaceuticals' (Burnaby, BC, Canada) systemic delivery program Apo-B SNALP completed its phase I study, providing some of the earliest systemic delivery results with an RNAi therapeutic. In total, 23 adult volunteers with mild hypercholesterolemia were enrolled, of whom 17 were exposed to Apo-B SNALP (Apo-B siRNA in an LNP formulation), the rest receiving placebo. Of three patients treated at the highest dose, 0.6 mg/kg, one experienced flu-like symptoms attributed to the siRNA; no other laboratory abnormalities (including changes in liver function tests) or safety events were reported. Interestingly, two of the patients on this dose patients showed reductions in circulating LDLc of 16.3% to 21.1% [71].

As would be expected, several RNAi therapeutic programs have been terminated. Bevasiranib (Opko Health Inc., Miami, FL, USA) is delivered via the intravitreal route and targets VEGF. In 2006, a randomized, double-blind, phase II study comparing three doses was completed patients with in wet AMD (*n *= 129) [72]. The safety data were encouraging, but the absence of a placebo arm prevented interpretation of any efficacy signals. Despite this limitation, and a report that its efficacy might be mediated via TLR-3 activation [14], 330 patients were enrolled in a phase III trial to study the safety and efficacy of bevasiranib in wet AMD [73]. Unfortunately, the study was terminated prematurely after the Independent Data Monitoring Committee reported that although safety was acceptable, the study was unlikely to reach its primary endpoint, an improvement in visual acuity [74]. Similarly, the intravitreal program AGN211745 (Allergan Inc., Irvine, CA, USA) which targets VEGFRI for wet AMD, was also terminated in phase II [75] after initial positive reports of efficacy from an earlier study [76]. AGN211745 too was shown to be possibly mediating its effects in preclinical models of AMD via TLR-3 activation [14].

From all the RNAi programs to date, only two, Bcr-abl and TD101 (Transderm Inc., Santa Cruz, CA, USA), are currently associated with completed safety and efficacy studies that have been published. However, both programs enrolled only one patient each. Koldehoff *et al*. [77] treated a single patient with recurrent CML by systemic administration of a formulated siRNA against Bcr-abl; however, the reported proof of concept with Bcr-abl knockdown in circulating leukemic cells is difficult to interpret because of the use of concomitant medications. Leachman *et al*. [78] reported a phase 1b study in a single patient with pachyonychia congenita, an autosomal dominant condition with painful palmoplantar calluses secondary to a keratin K6a mutation. The safety and efficacy of TD101 was tested in a 17-week, prospective, double-blind, split-body, vehicle-controlled, dose-escalation trial on a single patient. Randomly assigned solutions of TD101 or vehicle control were injected into symmetric plantar calluses on the opposite feet. No adverse events occurred during the trial or in the 3-month washout period. Subjective patient assessment and physician clinical efficacy measures revealed regression of callus on the siRNA-treated, but not on the vehicle-treated foot.

## Conclusions

With barely a decade since its initial characterization, the translation of RNAi biology toward RNAi therapeutics has progressed at a rapid pace. Moreover, the following years promise to be crucial for demonstration of the safety and efficacy of this new method in controlled, randomized studies. Certainly, much remains to be accomplished. In delivery research and preclinical studies, further advancement of the field will require continued progress in conjugation and formulation strategies. It would be naive to believe that any one single technology will provide all the solutions, so it is gratifying to see such a broad-based effort across multiple delivery technologies under investigation in academia and industry. In development efforts, many milestones remain to be achieved, including the conduct of large phase III studies and regulatory approvals. Of course, it is likely that there will be product failures, which could be accounted for by a number of factors including target selection, delivery technologies, clinical trial design and even commercial considerations. Nevertheless, the steadfast commitment in the field is apparent in the advancement of this critically needed innovation to patients.

## Competing interests

All authors are employees of Alnylam Pharmaceuticals Inc., Cambridge, MA, USA which is engaged in the discovery and development of RNAi therapeutics.

## Authors' contributions

The primary draft was generated by one of us (AKV) after many useful planning and guiding discussion with the other authors. All authors then contributed additional sections and helped with critical review and editing, leading to the final draft.
